# Effect of bioagents on cucumber seed mycoflora, seed germination, and seedling vigour

**DOI:** 10.1038/s41598-023-30253-3

**Published:** 2023-04-13

**Authors:** Aman Sharma, Arti Shukla, Meenu Gupta

**Affiliations:** 1 Department of Plant Pathology, Dr Y.S. Parmar University of Horticulture and Forestry, Nauni, Solan, Himachal Pradesh 173230 India; 2 HRTS & KVK Kandaghat, Dr Y.S. Parmar University of Horticulture and Forestry, Nauni, Solan, Himachal Pradesh 173213 India; 3 Department of Vegetable Science, Dr Y.S. Parmar University of Horticulture and Forestry, Nauni, Solan, Himachal Pradesh 173230 India

**Keywords:** Ecology, Plant sciences

## Abstract

The effect of different bioagents such as *Trichoderma harzianum, T. viride, T. virens, Pseudomonas fluorescens*, and *Bacillus subtilis* was studied on seed mycoflora, seed germination, root/shoot length, and seedling vigour of cucumber var. Solan Srijan under in vitro conditions. *Alternaria* sp., *Aspergillus* sp., and *Fusarium* spp. were observed on cucumber as seed mycoflora, with *T. harzianum* showing the greatest inhibition for *Alternaria* sp. and *Fusarium* spp., and *T. viride* showing the greatest inhibition for *Aspergillus* sp. Cucumber var. Solan Srijan seeds were treated with various bio agents, with *T. harzianum* being the most effective in increasing seed germination (88.75%), root length (13.58 cm), shoot length (14.58 cm), and seedling vigour (2501.31).

## Introduction

Cucumber (*Cucumis sativus* L.) is a widely cultivated plant in the cucurbitaceae family. It is native to India (presumably from the Himalayan foothills) and was domesticated around 3000 years ago^1^. Cucumber is a monoecious vine with coarse leaves, yellow flowers and rough stems. Cucumber is the 3rd most popular vegetable crop in the world after tomato and onion^2^. *Trichoderma* spp. are being used more widely in plant agriculture, both for disease control and yield enhancement^3^. Bharath et al*.* observed that seed treatment with antagonists like *Trichoderma harzianum* and *T. viride* improved seed germination, and seedling vigour and reduced the incidence of seed-borne fungal pathogens^4^. Mogle and Maske investigated the effect of various Leaf extracts alone and in combination with *Trichoderma* and fungicides on Cowpea seed mycoflora, germination, and vigour index^5^. Gawade et al.^6^ conducted an experiment in which seeds of mungbean cv. Vaibhav was treated with bioagents and botanicals, including talc powder formulations of *Pseudomonas fluorescens* (0.6%), *Trichoderma viride* (0.6%), *Pseudomonas fluorescens* + *Trichoderma viride* (0.6%) each, garlic extract (1%), ginger extract (1%) and Thiram + Carbendazim (0.2% each) as a recommended fungicidal check. Vandna and Priya^7^ investigated the efficacy of *Trichoderma harzianum* as a seed treatment against *Fusarium oxysporum* f. sp. *lycopersici* causing wilt disease of tomato and the results showed maximum seed germination (78.33%) and disease control (66.53%) with *T. harzianum* seed treatment. Biopriming with *T. harzianum* for 12 h had the highest germination percentage of 92.92%, followed by T13 treatment i.e. biopriming with *P. fluorescens* for 12 h with a germination percentage of 90.75%^8^. The study aimed to study the effect of different bioagents on seed mycoflora, seed germination, root/shoot length, and seedling vigour of cucumber var. Solan Srijan under in vitro conditions.

## Results

### Effect of seed treatment with bio agents on seed mycoflora

Germination of seeds was done over 8 days. *Alternaria* sp., *Aspergillus* sp. and *Fusarium* spp. were the most common pathogens found on cucumber seeds. The results (Table 1) revealed that all five test bio agents significantly inhibited seed mycoflora of *Alternaria* sp. as compared to the untreated control with per cent inhibition ranging from 89 to 95%. *T. harzianum*, on the other hand, inhibited mycoflora the most (95%) followed by *T. virens* (94%) while *T. viride* and *P. fluorescens* inhibited the seed mycoflora to the same extent (91%). *B. subtilis* was found to be the least effective (89%). The results (Table 1) revealed that all five test bio agents significantly inhibited seed mycoflora of *Aspergillus* sp. compared to the untreated control with per cent inhibition ranging from 87 to 95%. *T. viride* inhibited mycoflora the most (95%), followed by *T. harzianum* (93%), *T. virens* (92%), *P. fluorescens* (90%) and *Bacillus subtilis* (87%) in descending order. A perusal of the results (Table 1) revealed that all five test bio agents significantly inhibited seed mycoflora of *Fusarium* spp. compared to the untreated control with inhibition ranging from 87 to 96%. However, *T. harzianum* inhibited mycoflora the most (96%), followed by *T. virens* (94%) and *T. viride* (93%) while *Bacillus subtilis* (87%) was the least effective.

**Table 1:** Effect of seed treatment with bio agents on seed mycoflora.

Sr. no.	Treatment	Inhibition of mycoflora (%)		
		*Alternaria* sp.	*Aspergillus* sp.	*Fusarium* spp.
1.	*Trichoderma harzianum*	95 (77.21)	93 (74.76)	96 (78.43)
2.	*Trichoderma viride*	91 (72.58)	95 (77.21)	93 (74.76)
3.	*Trichoderma virens*	94 (75.98)	92 (73.54)	94 (75.98)
4.	*Pseudomonas fluorescens*	91 (72.58)	90 (71.62)	89 (70.66)
5.	*Bacillus subtilis*	89 (70.66)	87 (68.87)	87 (68.87)
6.	Control	80 (63.46)	82 (64.90)	80 (63.46)
	CD_(0.05)_	(3.37)	(2.90)	(3.11)

Figures in the parentheses are arc sine transformed. Statistical analysis was done on opstat software.

### Effect of bio agents on seed germination and seedling vigour of cucumber

The results showed that all three *Trichoderma* spp. tested were effective in increasing the percentage of germination (Table 2). However, among the three species, *T. harzianum* (88.75%) significantly improved the germination percentage in cucumber seeds. In comparison to the control (62.50%), *T. viride* (82.50%) and *T. virens* (85.00%) efficiently improved seed germination. Seeds treated with *Pseudomonas fluorescens* (72.50%) and *Bacillus subtilis* (68.75%) germinated at a higher rate than control seeds. Overall cucumber seed germination percentage was highly increased by *T. harzianum* and least increased by *Bacillus subtilis* as compared to the control seeds. Bio agents produced considerably longer shoot and root lengths than the control (untreated) (Table 2). In comparison to other bio agents, *Trichoderma harzianum* has the greatest potential to induce seedling root (13.58 cm) and shoot (14.58 cm) elongation. *Trichoderma virens* and *Trichoderma viride* were next with roots (13.00 cm, 12.06 cm) and shoots (13.86 cm, 13.26 cm) of different lengths, respectively. *Bacillus subtilis* had the shortest seedling length with a root and shoot length of 10.43 cm and 11.77 cm, respectively. Among the bio agents, the maximum seedling vigour index was observed in seed treatment with *Trichoderma harzianum* (2501.31) followed by seed treatment with *Trichoderma virens* (2286.19) and the minimum vigour index was observed in seed treatment with *Bacillus subtilis* (1523.25) over untreated seeds having vigour index of 1100.75 (Table 2).

**Table 2:** Effect of bio control agents on shoot length, root length, germination percentage and seedling vigour of cucumber var. Solan Srijan.

S. no.	Treatment	Seed germination (%)	Mean shoot length (cm)	Mean root length (cm)	Seedling vigour
Fungal bio agents					
1.	*Trichoderma harzianum*	88.75 (70.44)	14.58	13.58	2501.31
2.	*Trichoderma viride*	82.50 (65.29)	13.26	12.06	2089.13
3.	*Trichoderma virens*	85.00 (67.33)	13.86	13.00	2286.19
Bacterial bio agents					
4.	*Pseudomonas fluorescens*	72.50 (58.37)	12.43	11.89	1765.35
5.	*Bacillus subtilis*	68.75 (56.03)	11.77	10.43	1523.25
6.	Control	62.50 (52.22)	8.01	9.50	1100.75
	CD_(0.05)_	(3.65)	1.64	1.01	212.81

Figures in the parentheses are arc sine transformed. Statistical analysis was done on opstat software.

## Discussion

The current findings are in agreement with those of Bharath et al*.*^4^; Kakde and Chavan^9^; Mogle and Maske^5^; Singh et al.^10^; Gawade et al*.*^6^, and Kumari^11^ who reported T. harzianum's antagonistic activity against seed mycoflora. According to Kumari^11^, *Trichoderma* species have antagonistic activity against storage pathogens and contaminants such as *Aspergillus* spp., *Penicillium* spp. and *Alternaria* spp. Singh et al*.*^10^ reported that *Trichoderma harzianum*, *Pseudomonas fluorescens*, *Bacillus subtilis*, *T. virens* and *T. viride* showed promising results against seed-borne fungi. Several cell wall degrading enzymes, such as chitinase and glucanase, play an important role in *Trichoderma*'s antagonistic action against a wide range of fungal pathogens.

In the earlier studies, it was found that *T. harzianum* and *T. viride* have improved seed germination, root length and shoot length^12^. In the current study seeds treated with different bioagents had more seedling vigour and seed germination as compared to untreated seeds which means these treatments improve the seedling quality for growers. Seeds pretreated with *Trichoderma viride*, *Trichoderma harzianum* demonstrated enhanced seed germination rates and seedling vigour when compared to control seeds^13,14^. Kumari^11^ found that *Trichoderma harzianum* seed treatment resulted in the highest vigour index. The results of the current study also corroborate the findings of other researchers^8,15–18^.

## Methods

### Effect of seed treatment with bioagents on seed mycoflora

#### Collection of plant material

The seeds of the Solan Srijan variety of cucumber were bought from the Department of Seed Science and Technology Nauni, Solan (HP). The variety is a prolific fruit bearer that matures 55–60 days after planting. Fruits are cylindrical, crispy, and green in colour, measuring 18–22 cm in length and weighing 255–265 g on average. It produces approximately 10–15 fruits/plants. The average yield per acre is 200–225 q/ha. It is suitable for cultivation in Himachal Pradesh and other hilly states.

#### Seed treatment

Seeds with no cracks or othesr visible deformations were selected and surface sterilised for 3–5 min in a 0.1% sodium hypochlorite solution. Subsequently, the seeds were rinsed three times with sterile distilled water, air dried, and soaked for 4 h in *Trichoderma* spp*.* (10^6^ cfu/ml) and bacterial bioagents(10^8^ cfu/ml) broth. The details of bio agents used for seed treatment are given in Table 3.

**Table 3:** Details of bioagents used in the study.

Sr. no.	Bioagent	Source
1.	*Trichoderma harzianum*	Department of Plant Pathology, YSP UHF, Nauni, Solan (HP)
2.	*T. viride*	-do-
3.	*T. virens*	-do-
4.	*Pseudomonas flourescens*	Soil Microbiology Laboratory, Department of Soil Science and Water Management, YSP UHF, Nauni, Solan (HP)
5.	*Bacillus subtilis*	-do-

#### Seed mycoflora

The infestation of seed with mycoflora was observed using the standard Petri plate method, as recommended by ISTA^19^. Cucumber seeds were first treated with bioagents (Table 3) and then kept in Petri dishes (25 seeds per plate, 4 replications per treatment). These seeded plates were incubated at 25 °C for 7 days. Untreated seeds were used as control. The number of infected seeds was counted and the per cent inhibition of mycoflora was calculated using the following formula:

$${\text{Inhibition of mycoflora }}\left( \% \right) = \frac{{{\text{Total number of seeds }} - {\text{Number of infected seeds}}}}{{\text{Total number of seeds}}} \times 100$$

### Effect of bioagents on seed germination and seedling vigour of cucumber

Seeds of cucumber variety Solan Srijan were treated with different bioagents (Table 3) as mentioned earlier.

### Effect on seed germination (%)

According to ISTA, one hundred seeds per replication for each treatment were used to conduct the germination test^20^. This was done in the seed germinator at 25 °C using the paper roll and blotter paper method. After 4 and 8 days, the first and final counts were taken. The following formula was used to calculate the percentage of germination:

$${\text{Germination}}\left( \% \right) = \frac{{{\text{Number}}\;{\text{of}}\;{\text{seeds}}\;{\text{germinated}}}}{{{\text{Total}}\;{\text{number}}\;{\text{of}}\;{\text{seeds}}\;{\text{used}}}} \times 100$$

### Effect on mean shoot length (cm)/mean root length (cm)

Ten normal seeds were chosen randomly during the final count on the 8th day. Their root and shoot lengths were measured in centimetres (cm) using a common ruler for all the seedlings.

### Effect on seedling vigour

Seedling vigour was calculated as per the formula given by Abdul-Baki and Anderson^21^.

$${\text{Seedling vigour }} = {\text{ Germination }}\left( \% \right) \, \times \, \left[ {{\text{mean shoot length }}\left( {{\text{cm}}} \right) \, + {\text{ mean root length }}\left( {{\text{cm}}} \right)} \right]$$

### Statistical analysis

All the experiments were carried out in a completely randomized design (CRD) with four replications. The collected data were subjected to one-way ANOVA. The significance of treatment means was practiced at a 5% level of probability^22^. Statistical analysis was done on OPSTAT software for the experiment (http://14.139.232.166/opstat/ ).

### Research ethics

Experimental research on seeds/plants, including the collection of seed/plant material, complied with relevant institutional, national and international guidelines and legislation. Prior permission was undertaken from the Director of Research, Dr Y.S. Parmar University of Horticulture and Forestry, Solan, India.

## Data Availability

This manuscript does not have any supplementary data and original data will be provided by the corresponding author when requested.
